# Guilt, Shame and Academic Misconduct

**DOI:** 10.1007/s10805-023-09480-w

**Published:** 2023-05-08

**Authors:** Guy J. Curtis

**Affiliations:** School of Psychological Science, University of Western Australia, 35 Stirling Hwy, Perth, WA 6009 Australia

**Keywords:** Moral Emotions, Shame, Guilt, Academic Misconduct, Academic Integrity, Academic Dishonesty

## Abstract

Moral and self-conscious emotions like guilt and shame can function as internal negative experiences that punish or deter bad behaviour. Individual differences exist in people’s tendency to experience guilt and shame. Being disposed to experience guilt and/or shame may predict students’ expectations of their emotional reactions to engaging in immoral behaviour in the form of academic misconduct, and thus dissuade students from intending to engage in this behaviour. In this study, students’ (*n* = 459) guilt and shame proneness, their expectations of feeling guilt and shame if they engaged in academic misconduct, and their intentions to engage in academic misconduct were measured. Three of the four facets of the guilt and shame proneness scale [GASP: Guilt–Negative-Behavior-Evaluation (NBE), Guilt-Repair, Shame–Negative-Self-Evaluation (NSE)] had significant negative correlations with academic misconduct intentions, and these relationships were mediated by anticipating shame and guilt related to engaging in academic misconduct. These results suggest that for some students expecting to experience negative moral emotions when engaging in academic misconduct may protect them from breaching ethical assessment rules.

Former US President Donald Trump’s lack of shame over actions that other people would perceive as shameful, such as lying, has been repeatedly pointed out in the press both during (e.g., Rotner, 2019) and after (e.g., Garside, 2022) his term in office. Trump has been described as a habitual cheat when playing golf (Reilly, 2019) and, according to his sister, he cheated routinely on assessments when he was a college student (Trump, 2020). Could Trump’s shamelessness have anything to do with his willingness to cheat? Research has established that individual difference in personality and attitudes (e.g., Lee et al., 2020), as well as moral emotions (e.g., Curtis et al., 2022a), may be related to students’ engagement in academic misconduct. However, previous research has been equivocal as to whether personality-based individual difference in students’ propensity to experience the moral emotions of shame and guilt may be related to their intention to engage in academic misconduct (e.g., McTernan et al., 2014; Stanculescu, 2013). A possible explanation of these inconsistent results is that situation-specific anticipation of experiencing shame and guilt as a result of engaging in academic misconduct may mediate the relationship between dispositional shame and guilt proneness and academic misconduct intentions. To date, no previous study has tested this possibility and the study that is reported in this paper addresses this gap in the literature.

## Background

Academic misconduct is a violation of rules of academic integrity that can come in many forms including plagiarism and cheating (Eaton, 2021). Academic misconduct may be reduced by assessment security measures that make assessments less “cheatable”, such as by proctoring exams (Dawson, 2021). However, the ingenuity of students who are intent on cheating means that although some assessment types make cheating more difficult, no assessments are perfectly immune from cheating (Dawson, 2021). Because of this, moral, social, and educational factors that support academic integrity are important barriers to academic misconduct beyond assessment security, which can restrain students from cheating even when cheating may not only be possible, but easy (Eisenberg, 2004).

Despite barriers like assessment security limiting students’ engagement in academic misconduct, it is still a common occurrence. For example, a recent survey of over 1000 students at an Australian university indicated that around two-thirds of them engaged in some form of plagiarism (Curtis & Tremayne, 2021). In addition, the rapid transition to online teaching since the onset of COVID-19, along with insecure online assessment, has seen an increase in the use of online services associated with cheating (Hill et al., 2021; Lancaster & Cotarlan, 2021). With assessments such as unproctored online tests, students may easily look up answers, collude with other students, find answers using an artificial intelligence content generator like ChatGPT, or pay a “homework help” site to provide them with solutions to problems (Dawson, 2021). However, only some students who have these opportunities to engage in academic misconduct will do so.

Criminological theories can help to explain situations where opportunities for wrongdoing exist, but these opportunities are not necessarily taken up by the people they are presented to. For example, if a person leaves their wallet on a table at a café someone may walk past and steal it. Some people may steal it brazenly as they are watched by the owner of the wallet, others sneakily if the owner is looking away, but others still would not think of touching someone else’s wallet, even if the owner went to the bathroom and left it unattended. The rational choice perspective from criminology suggests that people weigh the costs, benefits, and risks when deciding whether to undertake an unethical action (Cornish & Clarke, 1987). For some people, the choice to steal a wallet may be rational because they perceive the benefit to themselves as high and the risks as low (they really need money and think they can outrun the owner), whereas for others the benefits do not outweigh the risks. The rational choice perspective implies that some people will perceive risks and costs of an action like theft, or academic misconduct, as including the psychological cost of experiencing aversive moral emotions like shame and guilt (Nagy & Groves, 2021; Rundle et al., 2019).

## Guilt and Shame

Moral emotions such as guilt, shame, and sympathy serve important functions in guiding adaptive and beneficial behaviour, and in providing people with feedback on whether their behaviour aligns with, or breaches, important social rules (Sznycer et al., 2016). As a species, people are good at predicting what their emotional reactions to future events will be, and anticipated negative emotions protect people from taking actions that will make them feel bad (Gilbert, 2007). Anticipating shame and guilt, in particular, is likely to dissuade people from taking risky, harmful, or regrettable actions that may hurt or disadvantage others (Cohen et al., 2006; Sznycer et al., 2016). Thus, in the rational calculus of deciding whether to cheat or plagiarize to gain an unfair advantage in an educational assessment, students’ anticipated shame and guilt may factor into their intention to engage in misconduct. Indeed, Curtis et al. (2022a) found that anticipating experiencing guilt and shame reduced the extent to which students intended to engage in a specific form of academic misconduct, contract cheating. Importantly, however, they did not examine personality-based individual differences in the tendency to experience guilt and shame in their study.

Some people are more prone to experience, or expect to feel, guilt and/or shame than others. Cohen et al. (2011) developed a measure of guilt and shame proneness that assesses these individual differences; the Guilt and Shame Proneness Scale (GASP). The GASP recognizes a private vs. public distinction between guilt and shame (Cohen et al., 2011)^1^. Guilt is an emotion that may be felt when people transgress a moral rule, or harm another person, but the actions that broke the rule or inflicted harm are not known to other people (Sznycer, 2019). Shame may be felt when transgression of moral rules, or actions that harm others, are publicly exposed (Sznycer, 2019; Sznycer et al., 2016). In addition, the GASP also separates individual differences in the tendency to evaluate actions as eliciting guilt and shame (i.e., proneness to these emotions) from actions that may ameliorate feelings of guilt and shame. Thus, the GASP assesses the extent to which people perceive actions as guilt and shame inducing, as well as the extent to which they tend to withdraw because of shame or act to repair feelings of guilt. Research on the GASP has found that a tendency to experience guilt in response to private transgressions is related to the tendency to take actions to repair these feelings (Cohen et al., 2011). On the other hand, the tendency to experience shame in response to public transgressions and the tendency to withdraw related to shameful feelings or situations are relatively orthogonal (Cohen et al., 2011).

Shame and guilt proneness have been shown to reduce immoral and unethical behaviour. For example, people prone to experience guilt are less likely to engage in counterproductive work behaviours like slacking off or stealing stationary (Cohen et al., 2011, 2013), and people prone to experience shame perceive unethical behaviours more harshly (Arli et al., 2016). For this reason, shame and guilt proneness may be expected to reduce academically unethical actions like misconduct.

## Guilt, Shame, and Academic Misconduct

A small amount of past research has examined whether guilt and shame proneness may influence students’ engagement in academic misconduct, and this research has produced mixed results. In one study, guilt-proneness, but not shame-proneness, was significantly negatively related to academic cheating (Stanculescu, 2013). In an unpublished study, students with higher proneness to guilt and shame found it more difficult to come up with excuses to rationalize cheating (Rettinger et al., 2012 as cited in Rettinger 2017). However, Murdock et al. (2008), found that externalization (i.e., blaming cheating on others) more strongly predicted cheating than did guilt and shame proneness. Similarly, McTernan et al. (2014) found that other variables such as impulsivity were stronger predictors of cheating across a range of domains, including education, than guilt and shame proneness.

**Fig. 1:**
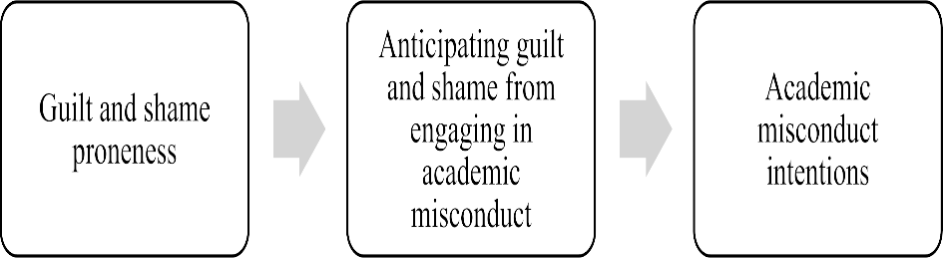
The expected relationship between guilt and shame proneness, anticipated guilt and shame from engaging in academic misconduct, and academic misconduct intentions

A possible reason for guilt and shame proneness not being strong predictors of academic misconduct in some previous studies may be the distinction between general and specific behavioural tendencies, attitudes, and intentions (Epstein, 1983). A *general* attitude against cheating, for example, may predict that students will generally cheat less across various settings, but it may not predict whether they will cheat on a *specific* test (e.g., Corey 1937). Similarly, a disposition, such as being introverted, suggests that a person may, in general, be shy, but does not guarantee that they will be tentative in every social situation they encounter (Locke et al., 2017). By the same token, proneness to feel guilt or shame may mean that a student is more likely to expect to feel guilty or ashamed if they cheat on an academic task. However, their specific expectation of feeling guilty or ashamed if they cheat on an academic task will better predict the student’s intention to cheat than their predisposition to experience guilt or shame per se. This expected relationship is displayed in Fig. 1.

## The Present Study

The present study tested whether anticipated guilt and shame related to specific acts of academic misconduct would mediate the relationship between guilt and shame proneness and academic misconduct intentions. Thus, in the present study students’ guilt and shame proneness was measured using the GASP scale. Next, they were presented with descriptions of several forms of academic misconduct behaviour. For each form of academic misconduct, students rated their intention to engage in the behaviour, their expectation of feeling guilt if they engaged in the behaviour undetected, and their expectation of feeling shame if they engaged in the behaviour and were caught having done so by their teacher.

The present study makes a new contribution to the literature by examining whether specific guilt and shame expectations related to academic misconduct may explain the potential for guilt and shame proneness more generally to predict academic misconduct intentions. The study specifically looked at *intentions* to engage in academic misconduct, rather than academic misconduct *behaviour* for several reasons. First, for volitional behaviour, intentions are a strong predictor of actual behaviour (Ajzen, 1991), and this is true of academic misconduct (e.g., Alleyne & Phillips 2011; Curtis et al., 2018). Evidence suggests that anticipated emotions can influence behaviour *because* they influence intentions, so intentions are an important step to assess in misconduct behaviour (Rivis et al., 2009). Furthermore, students in their early stages of higher education may have had few opportunities to engage in actual misconduct, but they are still able to report their plans to do so. Finally, for sensitive behaviours, such as academic misconduct, it is possible that people will under-report their behaviour (Curtis et al., 2022b).

The study tested the following hypotheses:

Given that some evidence exists for a relationship between guilt and shame proneness and reduced academic misconduct (e.g., Rettinger et al., 2012 as cited in Rettinger 2017), and this relationship is conceptually justified: H1. Guilt and shame proneness will be negatively correlated with academic misconduct intentions.

Given that specific attitudes and intentions tend to be positively related to general attitudes and intentions (Epstein, 1983): H2. Anticipating guilt and shame when engaging in academic misconduct will be positively correlated with guilt and shame proneness.

Given the results of Curtis et al. (2022a), who assessed anticipated guilt and shame related to engaging in contract cheating, without measuring guilt and shame proneness: H3. Anticipating guilt and shame when engaging in academic misconduct will be negatively correlated with academic misconduct intentions.

Given that specific attitudes tend to be more strongly related specific intentions than are general attitudes and dispositions (Epstein, 1983): H4. Anticipating guilt and shame when specifically engaging in academic misconduct will mediate the relationship between general guilt and shame proneness and academic misconduct intentions.

## Method

### Participants and Procedure

Data for this study were collected concurrently from the same participants as the study by Curtis et al. (2022a)^2^. Ethics approval to undertake this study was granted by the The University of Western Australia Human Research Ethics Committee. A sample of 621 higher education students were initially recruited through social media, paid or exchange recruitment platforms, and a psychology student research participation system. All students received either a small payment, academic credit, or the opportunity to enter a small prize draw in exchange for their participation. The final sample was 459, after students were removed for inattentive or incomplete survey responses. Most participants were Australian (397; 86.5%) they were most frequently studying psychology (30%; 18% health science, 16% science, 10% business/commerce, 9% arts/humanities), and ages ranged between 17 and 50 (*M*_*age*_ = 21.56; *SD*_*age*_ = 5.13).

The participants logged into an online survey using the Qualtrics survey system. They read an information form and provided consent to participate. Next, the measures in the study were presented in a random order for each participant, with demographic questions always presented last. Once the study measures were completed, the participants were redirected to a separate link to provide their identifying information for compensation and thanked for their participation.

### Measures

*Guilt and Shame Proneness* were measures using the Guilt and Shame Proneness Scale (GASP; Cohen et al., 2011). The GASP has 16 items in total and comprises four sub-scales of four items each: Guilt–Negative-Behavior-Evaluation (NBE), Guilt-Repair, Shame–Negative-Self-Evaluation (NSE), and Shame-Withdraw. Each item describes a scenario that may elicit guilt or shame or a response to a guilt or shame inducing situation. Participants then rated their likelihood of experiencing the emotion or taking the action described in the scenario on a 7-point scale from 1 = “Very Unlikely” to 7 = “Very Likely”. Subscale scores were formed by averaging the items in the subscales for each participant. An example item from the Guilt–Negative-Behavior-Evaluation (NBE) sub-scale is: “You secretly commit a felony. What is the likelihood that you would feel remorse about breaking the law?”, this sub-scale had a Cronbach’s alpha internal consistency of 0.68. An example item from the Guilt-Repair: “You reveal a friend’s secret, though your friend never finds out. What is the likelihood that your failure to keep the secret would lead you to exert extra effort to keep secrets in the future?”, this sub-scale had a Cronbach’s alpha internal consistency of 0.61. An example item from the Shame–Negative-Self-Evaluation (NSE) sub-scale is: “You give a bad presentation at work. Afterwards your boss tells your coworkers it was your fault that your company lost the contract. What is the likelihood that you would feel incompetent?”, this sub-scale had a Cronbach’s alpha internal consistency of 0.70. An example item from the Shame-Withdraw sub-scale is: “A friend tells you that you boast a great deal. What is the likelihood that you would stop spending time with that friend?”, this sub-scale had a Cronbach’s alpha internal consistency of 0.62. The sub-scales alpha internal consistencies in the present study that were consistent with those reported by Cohen et al. (2011).

*Anticipated guilt and shame related to academic misconduct, and academic misconduct intentions* were measured using a scale designed for this study. Students were presented with eight forms of academic misconduct,, with each defined and accompanied by a concrete example. This measure was based on the plagiarism measure developed by Maxwell et al. (2008) including shame paraphrasing, illicit paraphrasing, other plagiarism, purloining, but added four additional misconduct types: sham primary citation, blackmail, unauthorised additional test time, misrepresenting assignment length.

As an example item, the term **Sham Paraphrasing** was presented (in bold text) follow on the next two lines by:

#### “**Definition**

Material copied verbatim from text and source acknowledged (cited) in-line but represented as paraphrased not as quoted.

#### **Example**

A student copies a sentence directly from a journal article into his assignment. The student writes the name of the author and date of publication in brackets after the sentence but does not include quotation marks or a page number.”

For each form of academic misconduct students used a slider scale from zero to 100 in the Qualtrics online survey system to respond to the following questions: “How likely are you to intentionally engage in this kind of breach of academic integrity? (intention: 0 = “I would never do this” − 100 = “I am very likely to do this”); “How guilty would you feel if you engaged in this academic integrity breach and got away with it?” (anticipated guilt: 0 = “I would not feel guilty” − 100 = “I would feel extremely guilty”); “How much shame would you feel if you engaged in this academic integrity breach and got caught by your teacher?” (anticipated shame: 0 = “I would not feel ashamed” − 100 = “I would feel extremely ashamed”). The slider scale required students to move an arrow from the starting point (50) to indicate their response; if the arrow was unmoved a “missing” score was recorded. The ratings for intentions for the eight forms of academic misconduct were combined and averaged for each student. Anticipated shame and guilt were aggregated and averaged in the same way. For these newly-created scales, Cronbach alpha internal consistencies were satisfactory: intentions α = .77, anticipated guilt α = .86, anticipated shame α = .84.

## Results

### Data Screening

Data were assessed for compliance with statistical assumptions. All of the variables were sufficiently normally distributed to undertake parametric tests, with skew (all <+/-1.5) and Kurtosis (all < 3.5) well within acceptable bounds. Under 1% of data were missing completely at random (MCAR χ^2^(13,894) = 13900.25, *p* = .483) and these were replaced using expectation maximization.

### Correlations

Pearson’s product movement correlations among the variables of interest in the study are shown in Table 1. As shown in Table 1, all variables in the study correlated significantly with each other except for Shame-Withdraw and academic misconduct intentions. These results mostly support H1 and H3, as academic misconduct intentions were negatively correlated with anticipated shame and guilt, and three of the four facets of the GASP. Moreover, supporting H2, anticipation of shame and guilt in relation to engaging in academic misconduct were positively corelated with shame and guilt proneness.

Table 1 also shows the descriptive statistics for the measures. Interestingly, the mean anticipated shame (over 80 on a 100-point scale) that students expected if they were caught engaging in academic misconduct suggests a ceiling effect. Mean anticipated shame was significantly higher than mean anticipated guilt (*t*[458] = 21.97, *p* < .001). In contrast, mean academic misconduct intentions were relatively low, less than 20 on the 100-point scale.

**Table 1:** *Descriptive statistics and Pearson’s r correlations*

	*M* (*SD*)	1	2	3	4	5	6
1. Guilt-NBE	5.58 (1.13)						
2. Guilt-Repair	5.78 (0.91)	**0.54**					
3. Shame-NSE	6.00 (0.91)	**0.55**	**0.51**				
4. Shame-Withdraw	3.21 (1.14)	**0.10**	**0.10**	**0.11**			
5. Anticipated guilt	67.35 (19.43)	**0.56**	**0.37**	**0.45**	**0.14**		
6. Anticipated shame	80.47 (15.44)	**0.43**	**0.35**	**0.51**	**0.15**	**0.75**	
7. Academic misconduct intentions	19.60 (14.41)	**− 0.37**	**− 0.28**	**− 0.30**	0.09	**− 0.61**	**− 0.53**

*n* = 459, *p* < .05 in **bold**.

### Mediation Analyses

To test whether anticipated shame and guilt mediated the relationship between guilt and shame proneness and academic misconduct (H4), four mediation analyses were calculated in SPSS using Hayes’s (2017) Process Macro 4.2. The collinearity assumptions of regression were examined and no breaches were observed (all VIFs < 2.8). For the analyses, using Process model 4, the four sub-scales of the GASP were used as the separate predictor variables, anticipated guilt and shame were both entered as potential mediators, and academic misconduct intentions was the criterion variable. Mediation was assessed by examining boot-strapped confidence intervals for indirect effects.

The mediation analysis (see Table 2) revealed that for three of the GASP measures (Guilt-NBE, Guilt-Repair, Shame-NSE) their relationship with academic misconduct intentions were fully mediated by students’ anticipated guilt and shame associated with engaging in academic misconduct. Interestingly, Shame-Withdraw, while not significantly correlated with academic misconduct intentions, directly predicted academic misconduct intentions when anticipated guilt and shame were included in the analysis. Moreover, Shame-Withdraw positively predicted academic misconduct intentions, and anticipated guilt and shame only partially mediated this relationship. Of additional interest, the size of the indirect effects for anticipated guilt were higher in each of the four analyses than the indirect effects of anticipated shame.

**Table 2:** *Direct and indirect effects of guilt and shame proneness on academic misconduct intentions*

		Anticipated Guilt	Anticipated Shame
	Direct Effect (CI)	Indirect Effect (CI)	Indirect Effect (CI)
Guilt-NBE	− 0.57 (-1.68; 0.54)	-3.28 (-4.34; -2.25)*	− 0.88 (-1.65; − 0.14)*
Guilt-Repair	− 0.78 (-2.03; 0.47)	-2.78 (-3.82; -1.84)*	− 0.85 (-1.62; − 0.11)*
Shame-NSE	− 0.06 (-1.28; 1.40)	-3.45 (-4.63; -2.45)*	-1.31 (-2.39; − 0.29)*
Shame-Withdraw	2.34 (1.44; 3.24)*	− 0.88 (-1.56; − 0.29)*	− 0.35 (-0.74; -0.07)*

*n* = 459, * = confidence interval does not cross zero.

As a follow-up analysis, the four mediation models were re-run with students’ age and gender entered as covariates as research suggests that academic misconduct is more common among male than female students, and younger students as compared with older students in higher education (Bretag et al. 2019). As shown in Table 3, the significance (or non-significance) of all direct and indirect effects was not changed by contolling for these covariants.

**Table 3:** *Direct and indirect effects of guilt and shame proneness on academic misconduct intentions – controlling for age and gender*

		Anticipated Guilt	Anticipated Shame
	Direct Effect (CI)	Indirect Effect (CI)	Indirect Effect (CI)
Guilt-NBE	− 0.58 (-1.90; 0.39)	-3.68 (-4.52; -2.90)*	− 0.73 (-1.48; − 0.07)*
Guilt-Repair	− 0.68 (-2.16; 0.49)	-2.47 (-3.46; -1.58)*	− 0.72 (-1.51; − 0.02)*
Shame-NSE	− 0.06 (-1.33; 1.46)	-3.13 (-4.29; -2.16)*	-1.12 (-2.22; − 0.06)*
Shame-Withdraw	2.60 (1.67; 3.54)*	− 0.62 (-1.24; − 0.03)*	− 0.23 (-0.59; − 0.03)*

*n* = 459, * = confidence interval does not cross zero.

## Discussion

Partially supporting H1, guilt and shame proneness correlated negatively with academic misconduct intentions for three of the four measures from the Guilt and Shame Proneness Scale (GASP). Supporting H2 and H3, anticipating guilt and shame when engaging in academic misconduct were positively correlated with guilt and shame proneness and negatively correlated with academic misconduct intentions. H4 was also supported as anticipating guilt and shame when engaging in academic misconduct mediated the relationship between guilt and shame proneness and academic misconduct intentions. However, this mediation was only partial for Shame Withdrawal, which also had a direct positive, rather than negative, effect on academic misconduct intentions. These results persisted when age and gender of participants was controlled in the analyses.

Previous studies have found relationships between guilt and shame proneness and academic misconduct (Stanculescu, 2013), as well as the ability of students to rationalize engagement in cheating (Rettinger et al., 2012 as cited in Rettinger 2017). However, the relationship between guilt and shame proneness and academic misconduct has been weak or explained by other variables in some studies (McTernan et al., 2014; Murdock et al., 2008). The present study adds to the existing literature by showing that academic-misconduct-specific guilt and shame anticipation can explain and strengthen the relationship between dispositional guilt and shame proneness and academic misconduct intentions.

An interesting finding to consider is that the specific subscale of Shame Withdrawal in the GASP had a positive direct effect on academic misconduct intentions in the mediation analysis. Shame Withdrawal represents the tendency to take action to repair feelings of shame by removing oneself from the people or social situation associated with the shameful action or event. Cohen et al. ( 2011) found that Shame Withdrawal was positively associated with unethical business decisions as well as workplace and general delinquency, whereas the other scales of the GASP were negatively associated with these behaviours. People who have a tendency to withdraw from shameful situations may do this as a coping mechanism that allows them to engage in socially-sanctioned actions but then attenuate the bad feelings that come from being revealed as having undertaken these actions. Specifically, in this study, students who were higher in Shame Withdrawal may expect to escape the feelings of shame that may come from their teachers finding that they have engaged in academic misconduct, therefore they perceive misconduct as being a less risky behaviour to undertake.

There are two complimentary theoretical explanations for why academic-misconduct-specific guilt and shame anticipation mediate the relationship between guilt and shame proneness and academic misconduct intentions. First, as outlined earlier, situation-specific dispositions or attitudes better explain behaviours than general dispositions or attitudes (Epstein, 1983). A proneness to feeling shame means that a student may be more likely to feel ashamed about cheating, but asking them more precisely about their expected shame in relation to being caught cheating more accurately predicts their intentions in that situation. Second, the rational choice perspective suggests that people make a calculation based on the risks and rewards of an action that is, to some extent, shaped by their personal dispositions (Cornish & Clarke, 1987). For some students, anticipating feeling negative emotions such as shame from being caught cheating will figure into the calculation of the potential benefit of cheating and the risk of being caught. As noted above, a student who expects to manage shame by removing themselves from the shameful situation may calculate that shame is an acceptable risk of engaging in misconduct because they will overcome this feeling should it occur.

According to evolutionary psychology perspectives, self-conscious moral emotions serve an adaptive social function (Sznycer, 2019). Feeling pride is a self-rewarding emotion that makes people feel good about doing something that is good for their status (Sznycer, 2019). In contrast, guilt and shame discourage behaviours that undermine group rules, norms, safety, and morals – as well as prompting actions to mitigate these feelings (Cohen et al., 2011; Sznycer et al., 2016). The rules of academic misconduct and the social sanctions for breaking them in an academic environment are usually clear, which would explain why students tended to report quite high levels of anticipated shame if they were caught engaging in misconduct. In contrast, however, students reported lower levels of anticipated guilt that might come from undetected breaches of academic conduct rules. Because guilt comes from breaking internalized rules or moral, students may anticipated feeling less guilt than shame if they have not fully committed to academic integrity. Thus, this finding may suggest that the prospect of being caught for academic misconduct serves an important deterrent function over and above personal morals.

An interesting finding in the present study was the stronger mediation by anticipated guilt rather than anticipated shame for the relationship between dispositional guilt and shame proneness and academic misconduct intentions. One explanation of this finding is that the statistical relationships with anticipated shame were weakened by restricted range as a consequence of the ceiling effect for this variable. Another possible explanation, however, is that for students with strong internalized moral positions against cheating and plagiarism, the prospective guilt associated with engaging in academic misconduct is particularly determinative of their intention to do so (Tatum, 2022). Thus, anticipated shame of being caught engaging in misconduct may act as a baseline of underlying worry for most students and the prospect of feeling guilty over and above this is an important further deterrent.

Attentive readers will have noticed that in the previous paragraph it is suggested that personal morals may underlie anticipated guilt, which adds a deterrent to cheating over and above the prospect of being caught, and in the paragraph before it is contended that detection of cheating may prompt shame, which adds a deterrent over and above personal morals. It is possible that both contentions are true. The results of the present study showed that both anticipated guilt and shame were significant mediators of the relationship between guilt and shame proneness and academic misconduct intentions. Ultimately, this means that both anticipated emotions matter, and importantly, as noted, both stem from different sources – privately vs. publicly straying from expected standards of behaviour. Practically then, these results suggest that educational institutions and educators should instill internal moral rules concerning academic integrity in students, e.g., via honor codes, but also have robust means of detection and sanctions for breaches of academic integrity rules. This practical suggestion aligns with many voices in the academic integrity literature who argue that there is no single solution to academic misconduct and that multiple barriers must be put in place to enhance academic integrity (e.g., Dawson 2021; Eaton, 2021; Rundle et al., 2020).

A finding of further interest in this study was the high correlation between the measures of anticipated guilt and shame related to breaching academic integrity. The measurement used in this study for anticipated guilt and shame related to academic misconduct captures the theoretical private vs. public distinction between these emotions. Students were asked about anticipated guilt related to getting away with misconduct and anticipated shame related to being caught by their teacher for misconduct. One explanation for the high correlation between anticipated guilt and shame measures is that it is possible that some participants did not grasp this distinction in the measure and consequently rated their anticipation of shame and guilt similarly for engagement in academic misconduct. However, anticipated shame ratings were significantly higher than anticipated guilt ratings, which suggests that the student participants did treat these ratings differently. An alternative explanation of the high correlation, then, may be that a third factor underlies students’ anticipated guilt and shame related to engaging in academic misconduct – for example, valuing of academic integrity. If the value students place on academic integrity informs their anticipated guilt and shame related to engaging in academic misconduct these ratings would covary even if the level of one (anticipated shame) is higher than the level of the other (anticipated guilt). This explanation of the relationship is consistent with Sznycer’s (2019) research and theory that the *value* to the person of a moral transgression underpins the strength of their moral emotions, and the consequence for the transgression may further strengthen the emotion. Consequently, emphasizing the importance of academic integrity to students, e.g., via honor codes, may help to calibrate their moral-emotional anticipated reactions to academic misconduct.

### Limitations

The present study collected data using a cross-sectional self-report design. Cross-sectional self-report designs only allow causality to be theoretically inferred, but they do not directly test sequential causal relationships. In particular, because anticipated guilt and shame were measured concurrently, the study cannot answer the question raised above of which of these adds to the other in predicting academic misconduct intentions. In the future, a longitudinal study design, where anticipated guilt and shame are measured serially would better delineate the relationships among the variables examined in this study. In addition, as noted in the introduction to the paper, there are sound justifications for examining academic misconduct intentions as a likely predictor of academic misconduct behaviour, and this is a common dependent variable in academic integrity research (e.g., Curtis et al., 2022a; Uzun & Kilis, 2020). Nonetheless, it is important to reiterate that academic misconduct behaviour was not directly examined in this study and therefore the results of the study speak specifically to how student contemplate their potential engagement in misconduct. In the present study, the measure used to assess academic misconduct intentions and anticipated guilt and shame produced a low mean intention (just under 20 on a 100-point scale). These low average ratings of intentions may be a consequence of the language used in the dependent measure reducing students’ willingness to report their intended actions. Krásničan et al. (2022) suggested that survey language that specifically describes behaviour as cheating (or, similarly, a breach of acceptable academic standards), may lead students to under-report engagement in such behaviour (or, similarly, intentions to engage in such behaviour). Under-reporting of misconduct intentions may have restricted the range of the intentions variable, and, statistically, restricted range may reduce the strength of correlations between variables as compared with the true strength within a population. Nevertheless, it is notable that the study mostly found significant and theoretically predicted correlations with the intentions variable as measured. Still, future studies should use measures of academic misconduct intentions that do not label the behaviour as a breach of academic integrity.

## Conclusion

As stated at the outset of this paper, Donald Trump is shameless and a cheat (Reilly, 2019; Rotner, 2019). The question was posed: is his shamelessness related to his cheating? This paper furthered the examination of the question of whether there is a relationship between moral emotions like shame and guilt and academic misconduct like cheating and plagiarism. The study reported in this paper found that guilt and shame proneness predict academic misconduct intentions, mediated by anticipating guilt and shame specific to engaging in, or being caught engaging in, misconduct. Anticipated guilt and shame, as well as guilt and shame proneness, predicted reduced academic misconduct intentions. However, students who tend to manage shame by withdrawing from shameful situations had higher academic misconduct intentions. So, to answer the earlier questions: yes, Trump’s shamelessness likely contributes to his cheating.

Guilt and shame are emotional responses that shape socially-adaptive behaviours by rebuking unhelpful or unacceptable behaviours. Theoretically, the basis of guilt is breaching internalized moral codes whereas the basis of shame is publicly breaching normative moral codes (Cohen et al., 2011; Sznycer, 2019). Anticipating guilt and shame can be a function of people’s predispositions to experience these emotions in general. However, the present study suggests that strengthening students’ anticipation of feeling guilt and shame about breaching academic integrity codes specifically may best reduce their intention to do so. Interventions such as honor codes and robust detection of, and consequences for, academic misconduct stand a good chance of leveraging these adaptive moral emotions to reduce students’ engagement in academic misconduct.

## Footnotes

1 Although it is outside the scope of this paper to discuss theories of moral emotions at length, interested readers may wish to explore the self-behaviour attributional theory of shame and guilt, e.g., Tangney (1990), which is sometimes contrasted with the public-private distinction used here (e.g., Cohen et al., 2011), and more recent evolutionary interpersonal-adaptionist models (e.g., Sznycer et al., 2019).

2 To be clear, this paper reports on the same students as Curtis et al. (2022a) but reports completely new data and measures not reported elsewhere.
